# Microcystin-LR Regulates Interaction between Tumor Cells and Macrophages via the IRE1α/XBP1 Signaling Pathway to Promote the Progression of Colorectal Cancer

**DOI:** 10.3390/cells13171439

**Published:** 2024-08-27

**Authors:** Xiaochang Wang, Yuechi Song, Xiaohui Lu, Hengshuo Zhang, Ting Wang

**Affiliations:** Department of Cell Biology, School of Basic Medical Sciences, Nanjing Medical University, Nanjing 210029, China; xiaochang@stu.njmu.edu.cn (X.W.); songyuechi@njmu.edu.cn (Y.S.); lvxiaohui222118@stu.njmu.edu.cn (X.L.); zhs18796304477@163.com (H.Z.)

**Keywords:** microcystin-LR, colorectal cancer, tumor microenvironment, macrophage, IRE1α/XBP1 signaling pathway

## Abstract

Microcystin-LR (MC-LR), a cyanobacterial toxin, is a potent carcinogen implicated in colorectal cancer (CRC) progression. However, its impact on the tumor microenvironment (TME) during CRC development remains poorly understood. This study investigates the interaction between tumor cells and macrophages mediated by MC-LR within the TME and its influence on CRC progression. CRC mice exposed to MC-LR demonstrated a significant transformation from adenoma to adenocarcinoma. The infiltration of macrophages increased, and the IRE1α/XBP1 pathway was activated in CRC cells after MC-LR exposure, influencing macrophage M2 polarization under co-culture conditions. Additionally, hexokinase 2 (HK2), a downstream target of the IRE1α/XBP1 pathway, was identified, regulating glycolysis and lactate production. The MC-LR-induced IRE1α/XBP1/HK2 axis enhanced lactate production in CRC cells, promoting M2 macrophage polarization. Furthermore, co-culturing MC-LR-exposed CRC cells with macrophages, along with the IRE1α/XBP1 pathway inhibitor 4μ8C and the hexokinase inhibitor 2-DG, suppressed M2 macrophage-induced CRC cell migration, clonogenicity, and M2 macrophage polarization. This study elucidates the mechanism by which MC-LR-mediated interactions through the IRE1α/XBP1 pathway promote CRC progression, highlighting potential therapeutic targets.

## 1. Introduction

Microcystins (MCs) are environmental toxins produced by blue-green algae, posing serious threats to aquatic ecosystems and the health of both humans and animals [1,2]. The cyclic heptapeptide MC has multiple structural variants, with MC-LR being one of the most abundant and toxic [3,4]. MC-LR is absorbed into the bloodstream through the gastrointestinal tract, affecting the intestines, liver, kidneys, reproductive system, cardiovascular system, and nervous system [5,6,7,8,9]. Previous studies have shown that MC-LR can induce ovarian inflammation in mice by activating cGAS-STING signaling pathway and promoting the production of inflammatory cytokines in granulosa cells [10]. MC-LR induces ferroptosis in brain cells through activation of the Erk/MAPK signaling pathway [11]. Long-term environmental-level MC-LR exposure leads to chronic colorectal inflammation, fibrosis, and barrier breakdown via the CSF1R/Rap1b signaling pathway [12]. As the primary organ responsible for absorbing MC-LR [13], the intestines require careful monitoring for its effects.

Colorectal cancer (CRC) has a high incidence and mortality rate worldwide [14,15]. Epidemiological studies and animal experiments have revealed a direct association between the development of CRC and MC-LR exposure [16,17,18]. Our earlier study indicated that MC-LR may enhance CRC cell migration and invasiveness by upregulating MMP13 expression [19]. Previous studies have shown that MC-LR promotes colorectal cancer progression by activating PI3K/Akt, Wnt/β-catenin, and SMAD2 signaling pathways [20,21]. The effect of MC-LR on tumor cells has been studied in previous research, with limited exploration into its effects on the complex tumor microenvironment (TME).

In the TME of most solid tumors, tumor-associated macrophages (TAMs) constitute the largest proportion of myeloid infiltrates [22,23,24]. Under different microenvironmental signals, macrophages can differentiate into the following subgroups with distinct functions: the pro-inflammatory phenotype M1 (classical activation) and the immunosuppressive phenotype M2 (alternative activation) [25,26]. Additionally, a high infiltration of TAM is linked to a worse prognosis in several tumors, including gastric cancer [27], breast cancer [28], CRC [29], liver cancer [30], and other malignancies [31].

Various factors can alter the tumor microenvironment (TME), including endoplasmic reticulum stress (ERS) [32,33]. Uncontrolled malignant cell proliferation creates a microenvironment characterized by nutrient limitations, high metabolic demands, acidosis, and hypoxia [34]. In this context, the protein folding capacity is impaired in the endoplasmic reticulum (ER), causing an enrichment of unfolded or misfolded proteins and the induction of ERS [34]. In mammalian cells, three ER transmembrane proteins function as sensors of ER stress, as follows: activating transcription factor 6 (ATF6), inositol-requiring enzyme 1α (IRE1α), and PRKR-like ER kinase (PERK) [35]. Under conditions of proteostasis, the molecular chaperone-binding immunoglobulin protein (BiP; also known as GRP78) binds to these sensors and keeps them in an inactive state [36]. During periods of ER stress, BiP exhibits a higher affinity for binding to misfolded or unfolded proteins, causing it to dissociate from the sensors. This event enables the activation and subsequent induction of the unfolded protein response (UPR; also known as the ERS response), which is an adaptive mechanism capable of restoring ER homeostasis through various mechanisms [34]. The IRE1α/XBP1 signaling pathway activates its ribonuclease activity under endoplasmic reticulum stress, splicing XBP1 mRNA to generate active transcription factor XBP1s, which can regulate the expression of multiple genes. Studies have demonstrated that increased tolerance to sustained ERS increases cancer cell survival [37], metastasis [38], angiogenesis [39], immune suppression [40], and drug resistance [41]. Additionally, MC-LR has been found to interact with GRP78, the key switch protein of ERS [42], and to activate pro-tumor pathways through ERS in liver cancer cells [43]. However, it is not clear how MC-LR regulates the TME in CRC through ERS.

In this study, we found that MC-LR promotes the recruitment of macrophages, activates ERS in CRC cells, and induces M2 polarization, thereby promoting the malignant progression of CRC. It is essential to investigate the potential association between MC-LR exposure and CRC, considering the poor prognosis of CRC. Identifying the impacts of MC-LR on the TME of CRC may help us to understand the MC-LR-mediated carcinogenic activity, and may benefit the treatment of CRC.

## 2. Materials and Methods

### 2.1. Cell Culture and THP-1 Polarization

The colorectal adenocarcinoma cell lines of DLD-1 and HCT116, along with THP-1, a human monocytic leukemia cell line capable of differentiating into macrophage-like cells, were sourced from the Type Culture Collection of the Chinese Academy of Sciences. These cells were maintained in RPMI-1640 medium (Gibco, Billings, MT, USA) supplemented with 10% Fetal Bovine Serum (Lonsera, Shuangru Biotechnology, Suzhou, China) and 1% streptomycin/penicillin solution (Gibco, Billings, MT, USA) within a humidified incubator set at 37 °C with 5% CO_2_. Regular testing for mycoplasma contamination was conducted using PCR, and all experiments utilized mycoplasma-free cells. The process for THP-1-induced macrophage polarization has been detailed in prior studies [44].

MC-LR (ENZO, Farmingdale, NY, USA) was dissolved in dimethyl sulfoxide (DMSO) to prepare a concentrated 1 mM solution. This stock solution was then diluted into the culture medium to obtain the desired concentrations.

### 2.2. Co-Culture of Macrophages and CRC Cells

The 6-well Transwell chamber was used to effectively segregate DLD-1 or HCT116 cells in the upper chamber (5.0 × 10⁵ cells/well) from M0 macrophages in the lower chamber (1.0 × 10⁶ cells/well). Following 48 h of MC-LR exposure, the cells were collected for further experimental analysis.

### 2.3. Cell Viability Assay

CCK-8 (Dojindo, Tokyo, Japan) was used to assess cell viability. 2-Deoxy-D-Glucose (2-DG, SJ-MN0028) and 4μ8C (SJ-MX0449) were obtained from Sparkjade Biotech (Qingdao, Shandong, China). 2-DG is a glucose analog that acts as a competitive inhibitor of glucose metabolism, inhibiting glycolysis via its actions on hexokinase. 4μ8C is a small-molecule inhibitor of IRE1α. Further details can be found in the Supplementary Information.

### 2.4. Cell Transfection and siRNA

A specific siRNA sequence (si-IRE1α: 5′–CGAUCGUGAAGCAGUUAGATT–3′; 5′- UCUAACUGCUUCACGAUCGTT–3′) was introduced into cells using the Lipofectamine™ RNAiMAX (Invitrogen, Carlsbad, CA, USA) in Opti-MEM^TM^ medium (Gibco, Billings, MT, USA), according to the manufacturer’s guidelines. The sequence was based on previous research [45]. After a transfection period of 6 h, the cells were exposed to MC-LR for 48 h and then collected for the following experiments. siRNAs in the study were chemically synthesized using Tsingke Biotechnology (Beijing, China).

### 2.5. Animal Experiments

Specific pathogen-free (SPF) male BALB/c mice, aged 4 to 5 weeks and weighing between 15 and 20 g, were obtained from GemPharmatech (Nanjing, Jiangsu, China), and were housed at the Animal Core Facility of Nanjing Medical University. The AOM/DSS mouse model is an animal model used in CRC, particularly in exploring the mechanisms of colitis-associated cancer. This model combines the use of azoxymethane (AOM) and dextran sulfate sodium (DSS). AOM is a chemical carcinogen known for its ability to cause DNA damage and mutations. DSS, on the other hand, acts as a chemical agent that induces inflammation and can lead to colitis in mice. This condition is marked by symptoms including bloody stools, ulcers in the intestinal mucosa, and infiltration of granulocytes. The approach employed in establishing the MC-LR-exposed AOM/DSS mouse model was consistent with prior studies [44]. The AOM/DSS model can successfully induce the formation of multiple adenomas or adenocarcinomas in the colons of mice, which is similar to the occurrence and development of CRC in humans. The animal protocols were monitored and approved by the Laboratory Animal Ethics Committee of Nanjing Medical University (Approval No. IACUC-2212034). Supplementary Information described the exact procedure.

### 2.6. Hematoxylin and Eosin (HE) Staining and Immunohistochemistry (IHC)

Hematoxylin and eosin (HE) staining and immunohistochemistry (IHC) were conducted as reported previously [44], and related methods were described in the Supplementary Information. IHC was performed using anti-GRP78 (ab21685; Abcam) and anti-CD31 (2867; Cell Signaling) antibodies.

### 2.7. Immunofluorescence (IF)

The samples underwent an initial process of deparaffinization and hydration, succeeded by antigen retrieval utilizing an EDTA antigen retrieval solution. To prevent the runoff of antibodies, the tissue sections were encircled. Subsequently, a blocking step with hydrogen peroxide was implemented, followed by serum blocking using bovine serum albumin. The procedure concluded with an overnight incubation with primary antibodies anti-GRP78 (ab21685; Abcam) and anti-CD206 (24595; Cell Signaling). After incubation, secondary antibodies were applied, and signal amplification was achieved using tyramide (TSA). Microwave treatment was then used to remove the primary and secondary antibodies. TSA is a high-density in situ labeling method for detecting target proteins or nucleic acids based on the catalytic activity of HRP. The key principle is that fluorescently labeled tyramine, when activated by HRP and H_2_O_2_, binds covalently to tyrosine residues around the target, while the primary and secondary antibodies are non-covalently bound. Microwave treatment was used to elute the first round of primary and secondary antibodies, leaving the fluorescently labeled tyramine attached to the target. For the detection of a second target, a new round of labeling was performed without concern for cross-reactivity between the antibodies of the first and second rounds. Multiple target labeling was achieved by using different fluorescent labels. By repeating the immunolabeling process, double fluorescence staining was accomplished using different fluorescent tyramines. Subsequently, DAPI nuclear staining and the quenching of autofluorescence were conducted, after which the samples were mounted. Digital slide scanning and analysis were conducted using Servicebio Technology (Wuhan, China).

### 2.8. Transcriptome Sequencing and Data Analysis

In the RNA sample preparations, an input quantity of 1 μg of RNA was employed for each sample (*n* = 6, each group). Exact methods and analysis were described in the Supplementary Information.

### 2.9. Real-Time Quantitative PCR (RT-qPCR)

Real-time quantitative PCR (RT-qPCR) was conducted in accordance with previously established protocols [44], and related methods were described in the Supplementary Information. Primers used are presented in Supplementary Table S1.

### 2.10. Western Blot

The Western blot analysis was performed in accordance with previously established protocols [44], and related methods were described in the Supplementary Information. Proteins were detected with antibodies against GRP78 (ab21685; Abcam), IRE1α (3294; Cell Signaling), XBP1s (40435; Cell Signaling), HK2 (2867; Cell Signaling), and β-actin (AC038; ABclonal).

### 2.11. Measurement of Lactate Production

A Lactic Acid Assay Kit (Abbkine, Wuhan, China) was used to measure the lactate levels. In brief, 50 µL of either serum or cell supernatant was mixed with 50 µL of Lactate Assay Buffer and 50 µL of Working Reagent. The absorbance at a wavelength of 450 nm was subsequently quantified after incubating at 37 °C for 30 min in the dark, utilizing a microplate reader (Molecular Devices, CA, USA).

### 2.12. Transwell Migration Assay

For the Transwell migration assay, 24-well Transwell (Corning, NY, USA) plates were used. HCT116 or DLD-1 were seeded in the upper chambers containing serum-free culture medium. M2 macrophages were cultured in the lower chambers containing complete medium. After cell seeding, conditioned media containing MC-LR (0.1 μM) was added to both the upper and lower chambers. This media was used alone or in combination with inhibitors (2-DG: 100 μM; 4-DG: 100 nM). After fixing the cells, staining them with crystal violet, and removing the cells from the upper chamber, random images of the bottom of the chamber were captured.

### 2.13. Colony Formation Assay

In cell colony formation assays, 600 HCT116 or DLD-1 were seeded in the lower chambers of 6-well Transwell plates, and M2 macrophages were placed in the upper chambers. The culture medium was supplemented with MC-LR (0.1 μM) alone or in combination with inhibitors (2-DG at 100 μM and 4μ8C at 100 nM). After an 8-day incubation, the cells were stained with 0.2% crystal violet solution for 30 min, and the number of cell colonies was quantified.

### 2.14. Statistical Analysis

Statistical results are presented as mean ± standard error of the mean (SEM), with all experiments performed in at least triplicate. Statistical analysis was conducted using GraphPad Prism software, version 9.0 (GraphPad, La Jolla, CA, USA), applying either the student’s *t*-test or one-way analysis of variance (ANOVA) to determine significance. A *p*-value of less than 0.05 was considered statistically significant.

## 3. Results

### 3.1. MC-LR-Promoted Malignant Progression of CRC in AOM/DSS Mice

**Figure 1:**
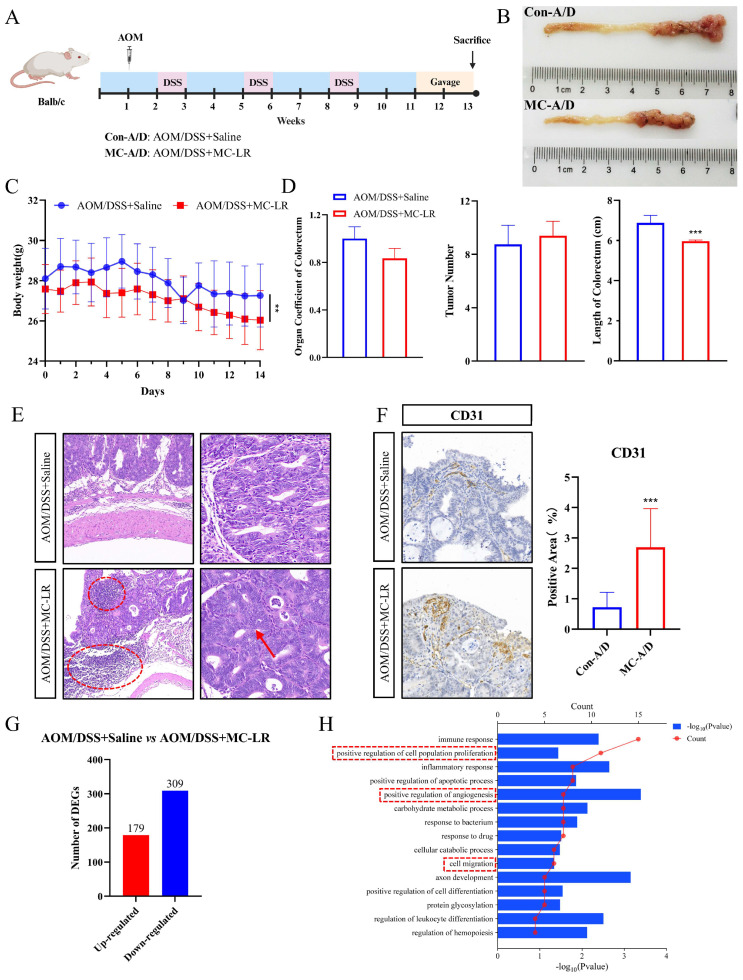
The facilitative impact of MC-LR on the development of AOM/DSS-induced CRC in mice. (**A**) Diagram illustrating the experimental procedure. (**B**) Example images of the colon and rectum. (**C**) Variation in body weight among AOM/DSS mice during MC-LR administration (*n* = 12 per group). (**D**) Colorectal organ coefficient, tumor count, and colon length in both the control and MC-LR-treated groups (*n* = 12 per group). (**E**) H&E staining of colorectal tumor regions in the control and MC-LR-treated groups. Scale bars represent 100 μm (left) and 50 μm (right). Red dotted circle means inflammatory cell infiltration. (**F**) IHC staining for the vascular endothelial marker CD31 in the control and MC-LR-treated groups. Scale bar represents 100 μm. (**G**) Number of significantly up- and downregulated genes between the control and MC-LR-treated groups (*n* = 6 per group). (**H**) GO analysis of upregulated DEGs in the biological process category for both the control and MC-LR-treated groups. *** *p* < 0.005.

We orally gavaged 40 μg/kg MC-LR to AOM/DSS-induced CRC mice for two weeks (*n* = 12 per group) (Figure 1A). This concentration has been reported to cause no obvious liver tissue damage in normal mice [46], a phenomenon we also observed in AOM/DSS-induced CRC mice treated with MC-LR (Figure S1A,B). Mice subjected to MC-LR gavage showed significant shortening of the colorectum (Figure 1B,D) and a declining trend in the colorectum-to-body weight ratio (Figure 1D), which may be related to inflammation and a disturbed intestinal barrier. Both groups exhibited weight reduction; however, weight loss was notably more pronounced in the MC-LR group (Figure 1C). The number of colorectal tumors between the two groups was not significantly different (Figure 1D). Further histopathological evaluation according to established criteria [47,48] revealed extensive inflammatory cell infiltration (red circle), loss of basal membrane polarity in tumor cells, gland fusion (red arrow), and increased vascular size in the tumor sites of the MC-LR group (Figure 1E,F). 

An RNA-Seq analysis on tumor tissues from CRC mice exposed to saline or MC-LR was conducted. Transcriptomic data revealed DEGs compared to the control group, with 488 genes showing significant changes (*p* < 0.05), including 179 upregulated genes and 309 downregulated genes (Figure 1G). Further GO analysis of the upregulated DEGs showed enrichment in positive regulation of cell population proliferation, positive regulation of angiogenesis, and cell migration within the biological process category in the MC-LR group (Figure 1H). In summary, the findings suggest that AOM/DSS mice exposed to MC-LR exhibit promoted progression of CRC.

### 3.2. MC-LR-Promoted Recruitment of Macrophages and M2 Macrophages’ Polarization in the TME

**Figure 2:**
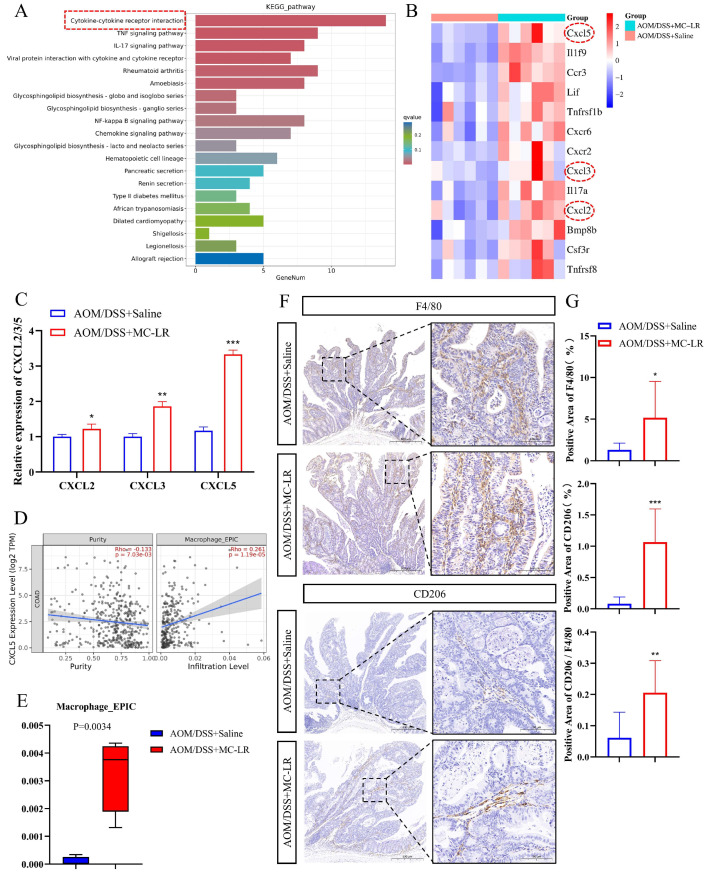
The enhancing influence of MC-LR on macrophage recruitment and M2 polarization in AOM/DSS-induced CRC mice. (**A**) KEGG pathway enrichment analysis for significantly altered pathways of DEGs in the control group compared to the MC-LR group. (**B**) Heatmap displaying DEGs involved in cytokine–cytokine receptor interactions. (**C**) RT-qPCR measurement of mRNA levels for CXCL2, CXCL3, and CXCL5 in both control and MC-LR groups (*n* = 4 per group). (**D**) Correlation analysis between CXCL5 expression and macrophage infiltration in colorectal adenocarcinoma (COAD), conducted using the TIMER 2.0 database. (**E**) Immune infiltration assessments of DEGs, as determined by the TIMER 2.0 database (*n* = 6 per group). (**F**) Representative IHC staining of F4/80 and CD206 in the control and MC-LR groups (*n* = 4 per group). Scale bar represents 100 μm. (**G**) IHC quantification of F4/80- and CD206-positive areas in both control and MC-LR groups. The percentage of M2 macrophages among total macrophages was calculated based on the positive area ratio of CD206 to F4/80. * *p* < 0.05, ** *p* < 0.01, *** *p* < 0.005.

KEGG pathway analysis of the upregulated DEGs revealed enrichment of cytokine–cytokine receptor interaction (Figure 2A). Within this pathway, comprising a total of 13 upregulated genes (Figure 2B), the expression of CXCL2 [49], CXCL3 [50], and CXCL5 [51] is associated with macrophage infiltration. RT-qPCR results showed increased expression of CXCL2, CXCL3, and CXCL5 in tumor tissues after MC-LR exposure (Figure 2C). Notably, CXCL5 expression in colon adenocarcinoma (COAD) and rectum adenocarcinoma (READ) correlated positively with macrophage infiltration (Figure 2D and Figure S2). Immune infiltration assessment of the DEGs revealed increased macrophage infiltration following MC-LR exposure (Figure 2E). IHC experiments using F4/80 (marker of macrophage) and CD206 (marker of M2 macrophage) in tumor tissues of the control and MC-LR groups showed elevated macrophage infiltration and an increased percentage of M2 macrophages after MC-LR exposure (Figure 2F,G). These findings collectively indicate that MC-LR may attract macrophages by enhancing CXCL5 expression in tumor cells and inducing their differentiation towards the M2 phenotype.

### 3.3. MC-LR-Induced IRE1α/XBP1 Pathway in CRC Cells to Promote Macrophage M2 Polarization

**Figure 3:**
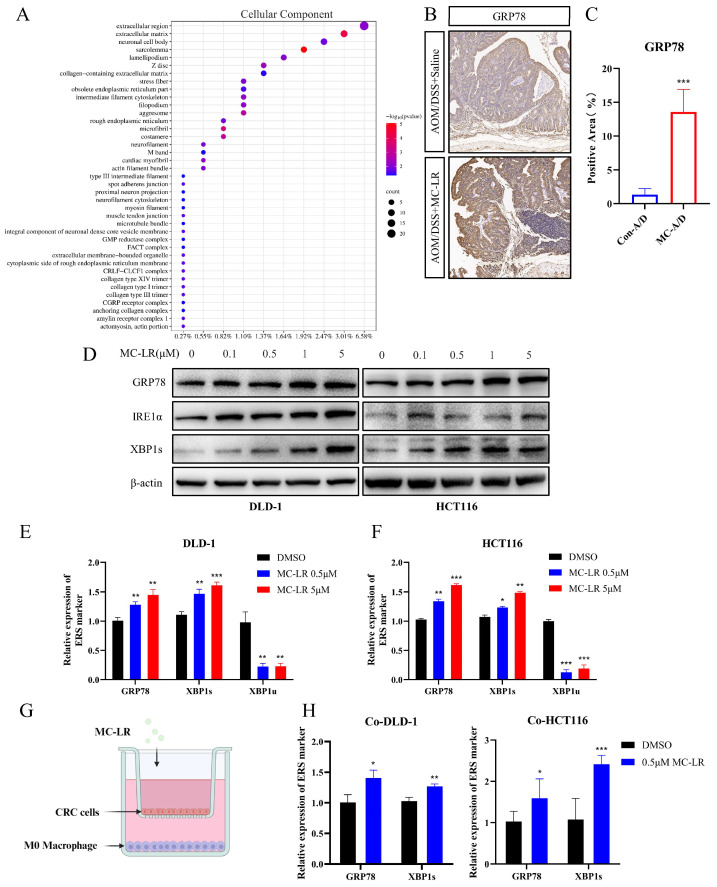
Induction of ERS by MC-LR in CRC cells. (**A**) GO analysis for cellular components comparing the control group with the MC-LR group. (**B**) Representative IHC staining of GRP78 in both the control and MC-LR groups. Scale bar, 100 μm (*n* = 4 per group). (**C**) IHC quantification of GRP78-positive areas in the control and MC-LR groups. (**D**) Protein levels of GRP78, IRE1α, XBP1s, and β-actin in DLD-1 and HCT116 cells after treatment with MC-LR (0, 0.1, 0.5, 1, 5 μM) for 48 h. Relative mRNA expression of GRP78, XBP1s, and XBP1u in (**E**) DLD-1 and (**F**) HCT116 cells after 48 h exposure to MC-LR (0, 0.5, 5 μM). (**G**) Schematic representation of the co-culture model (created using BioRender.com, https://www.biorender.com/). (**H**) Relative expression levels of GRP78 and XBP1s in HCT116 and DLD-1 cells co-cultured with M0 macrophages following 48 h exposure to MC-LR (0.5 μM). * *p* < 0.05, ** *p* < 0.01, *** *p* < 0.005. Original images can be found in Figure S1.

GO enrichment analysis of DEGs revealed multiple DEGs associated with endoplasmic reticulum function within the cellular component category (Figure 3A). Previous studies have confirmed that ERS can reshape the TME and promote tumor progression [52]. Therefore, we hypothesized that MC-LR induces ERS in CRC cells, thereby promoting M2 macrophage polarization. To confirm this hypothesis, IHC was carried out to identify the expression of ERS marker GRP78, and a significant increase was found in tumor tissues exposed to MC-LR (Figure 3B,C). Next, we treated CRC cells with different concentrations of MC-LR, as follows: 0.1 and 0.5 μM (non-cytotoxic concentrations), and 1 and 5 μM (near-cytotoxic concentrations) (Figure S3). The results revealed that MC-LR exposure increased GRP78 expression and activated the IRE1α/XBP1 signaling pathway, with the degree of activation increasing at higher concentrations in CRC cells (Figure 3D–F).

**Figure 4:**
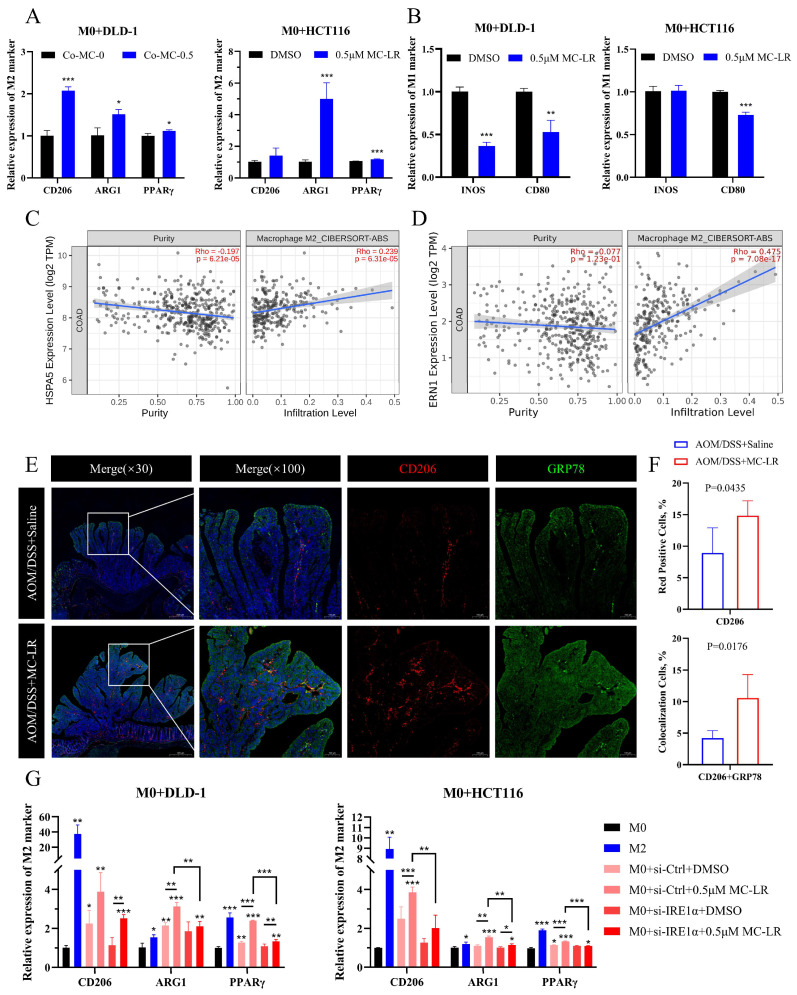
MC-LR-induced ERS in CRC cells facilitated the polarization of M0 macrophages towards the M2 phenotype. (**A**) Relative levels of M2 markers (CD206, ARG1, PPARγ) in M0 macrophages co-cultured with DLD-1 and HCT116 cells following 48 h exposure to MC-LR (0.5 μM). (**B**) Relative levels of M1 markers (CD80 and INOS) in M0 macrophages co-cultured with CRC cells (DLD-1 and HCT116) after 48 h of MC-LR (0.5 μM) exposure. The relationship between (**C**) GRP78 and (**D**) ERN1 (IRE1α) expression and M2 macrophage infiltration in COAD was analyzed using the TIMER 2.0 database. (**E**) Dual IF staining of GRP78 (green) and CD206 (red) in tumor tissues from the control and MC-LR-treated groups. Scale bar represents 100 μm. (**F**) Quantification of the positive area for CD206 (red) and the colocalization of CD206 with GRP78 (yellow) in tumor tissues from control and MC-LR groups. (**G**) Relative expression of M2 markers (CD206, ARG1, PPARγ) in M0 macrophages co-cultured with DLD-1 or HCT116 after 48 h exposure to MC-LR (0.5 μM), with IRE1α expression knocked down in HCT116 and DLD-1 cells using siRNA. * *p* < 0.05, ** *p* < 0.01, *** *p* < 0.005.

A Transwell co-culture model was established, with MC-LR (0.5 μM) added to both chambers (Figure 3G). RT-qPCR results indicated that the IRE1α/XBP1 signaling pathway was activated in CRC cells by MC-LR exposure (Figure 3H). M0 macrophages showed a tendency to polarize towards the M2 phenotype (Figure 4A) rather than the M1 phenotype (Figure 4B) under the co-culture conditions with MC-LR. Further analysis demonstrated a positive association between GRP78 and IRE1α expression and M2 macrophages’ infiltration in COAD and READ (Figure 4C,D; Figure S4A,B). Consistently, double label IF staining of GRP78 and CD206 in tumor tissues increased after exposure to MC-LR, corroborating the IHC results (Figure 4E,F). Knocking down the expression of IRE1α in CRC cells using si-RNA and then co-culturing with M0 macrophages showed that compared to the si-Ctrl DMSO group, the promotion of M2 polarization in the si-IRE1α DMSO group was weakened. However, compared to the si-IRE1α DMSO group, MC-LR exposure promoted M2 polarization in the si-IRE1α MC-LR group, although this promotion was lower than that observed in the si-Ctrl MC-LR group (Figure 4G). The results collectively indicate that MC-LR activates the IRE1α/XBP1 pathway in CRC cells to promote M2 polarization of macrophages.

### 3.4. MC-LR-Activated IRE1α/XBP1 Signaling Pathway Promoted M2 Macrophages’ Polarization through Lactate Secretion

**Figure 5:**
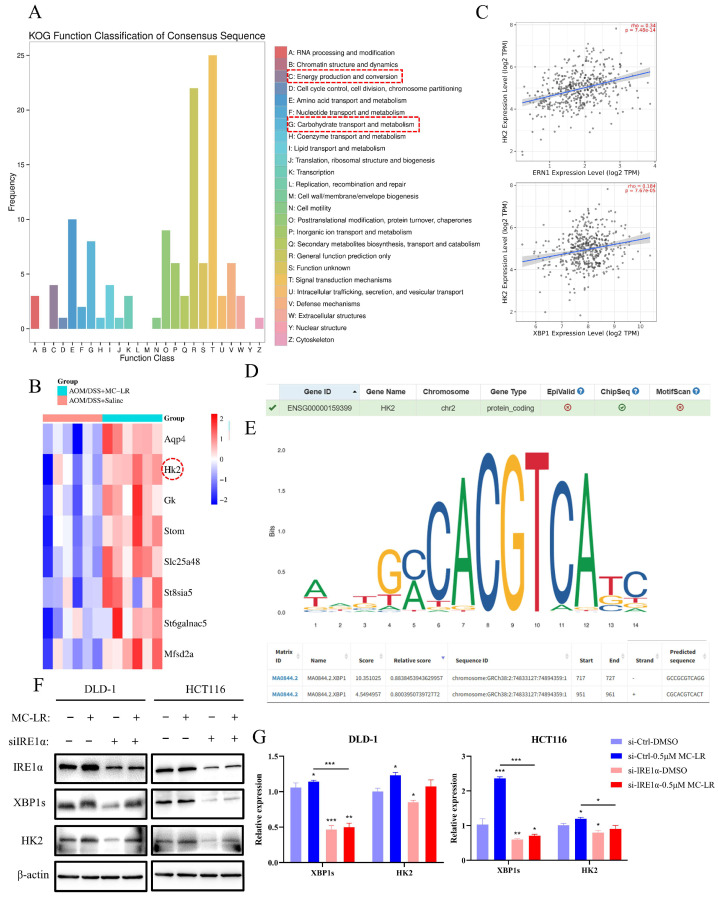
MC-LR-induced ERS upregulated the expression of HK2 in CRC cells. (**A**) KOG functional classification analysis of DEGs in the control and MC-LR-treated groups. (**B**) Heatmap showing DEGs related to energy production, conversion, and carbohydrate transport and metabolism. (**C**) Correlation between GRP78 and ERN1 (IRE1α) expression and HK2 expression in COAD analyzed using the TIMER 2.0 database. (**D**) HK2 was identified as a potential downstream transcriptional target of XBP1 via hTFtarget. (**E**) Prediction of XBP1 binding sites on the HK2 promoter region was performed using the JASPAR database (https://jaspar.genereg.net/, accessed on 15 April 2024). (**F**) Protein levels of IRE1α, XBP1s, HK2, and β-actin in HCT116 and DLD-1 following 48 h exposure to MC-LR (0.5 μM), with IRE1α expression knocked down via siRNA. (**G**) Relative mRNA expression of XBP1s and HK2 in DLD-1 and HCT116 after 48 h of MC-LR (0.5 μM) treatment, with IRE1α knocked down by siRNA. * *p* < 0.05, ** *p* < 0.01, *** *p* < 0.005. Original images can be found in Figure S1.

**Figure 6:**
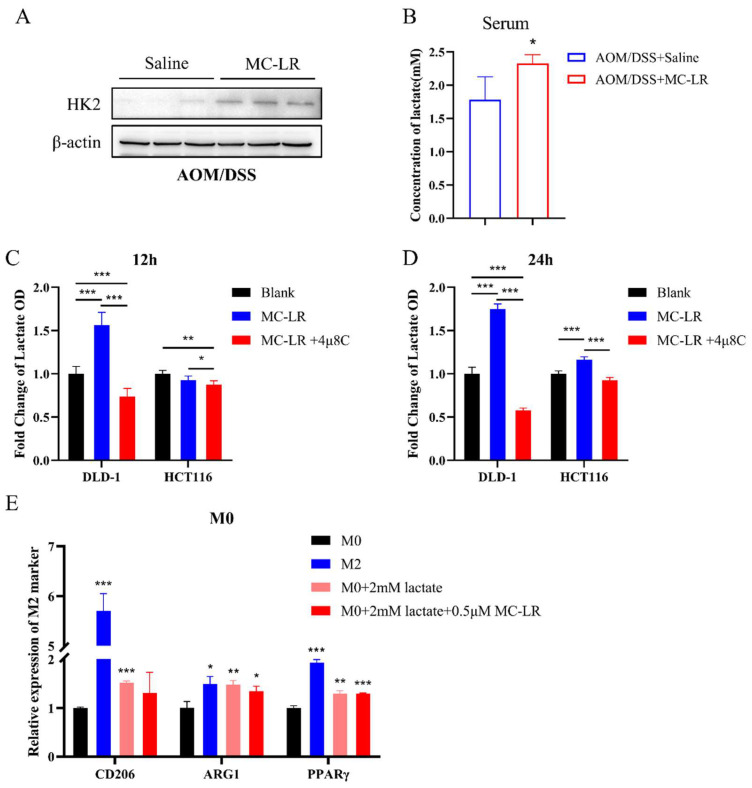
Lactate secretion, induced by ERS in CRC cells following exposure to MC-LR, facilitated M2 macrophage polarization. (**A**) Protein levels of HK2 and β-actin in the control and MC-LR-treated groups of AOM/DSS mice. (**B**) Lactate concentration in serum from control and MC-LR-treated groups of AOM/DSS mice. Fold change in lactate OD values in the culture medium supernatant of DLD-1 and HCT116 cells after treatment with MC-LR or MC-LR, combined with 4μ8C for (**C**) 12 h and (**D**) 24 h. (**E**) Relative expression of M2 markers (CD206, ARG1, PPARγ) in M0 macrophages following 48 h exposure to 2 mM lactate or a combination of MC-LR and 2mM lactate. * *p* < 0.05, ** *p* < 0.01, *** *p* < 0.005. Original images can be found in Figure S1.

KOG functional classification analysis of DEGs in tumor tissues emphasized energy production and conversion, as well as carbohydrate transport and metabolism (Figure 5A). Among the identified DEGs, HK2 expression was found to positively correlate with IRE1α and XBP1 in COAD and READ (Figure 5B,C and Figure S4C,D). After activation of the IRE1α/XBP1 pathway, transcription factor XBP1s could regulate the expression of numerous genes [53]. Using the hTFtarget website, we confirmed HK2 as a downstream target of XBP1s (Figure 5D). We obtained the HK2 promoter sequence from the UCSC database and used the JASPAR database to predict potential XBP1s’ binding sites on the HK2 promoter (Figure 5E). We then verified HK2 expression by Western blot and RT-qPCR in CRC cells exposed to MC-LR after knocking down IRE1α (Figure 5F,G). Compared to the si-Ctrl DMSO group, the expression of HK2 increased significantly in the si-Ctrl MC-LR group. Although HK2 expression decreased after knocking down IRE1α, it recovered following MC-LR exposure, but remained less than in the si-Ctrl MC-LR group. Moreover, the protein expression of HK2 in tumor tissues was elevated after MC-LR exposure (Figure 6A). Lactate is a primary product of glycolysis, as HK2 facilitates the initial and rate-limiting steps. Following MC-LR exposure, lactate concentration in the serum of CRC mice significantly increased (Figure 6B). In CRC cells, MC-LR treatment also increased lactate secretion into the culture medium, which was inhibited by using the IRE1α inhibitor 4μ8C during MC-LR exposure (Figure 6C,D). Next, we treated THP-1-induced M0 macrophages with lactate (2 mM), alone or together with MC-LR (Figure 6E). These results showed M2 polarization of macrophages after lactate treatment, with no significant effect of MC-LR on lactate-induced M2 polarization. These results collectively suggest that MC-LR-activated ERS promotes HK2 expression in CRC cells, enhancing lactate secretion in glycolysis. The secreted lactate further promotes M2 macrophage polarization.

### 3.5. Inhibition of IRE1α/XBP1 Pathway and Glycolysis Suppresses MC-LR-Mediated Malignant Progression of CRC Cells in TME

**Figure 7:**
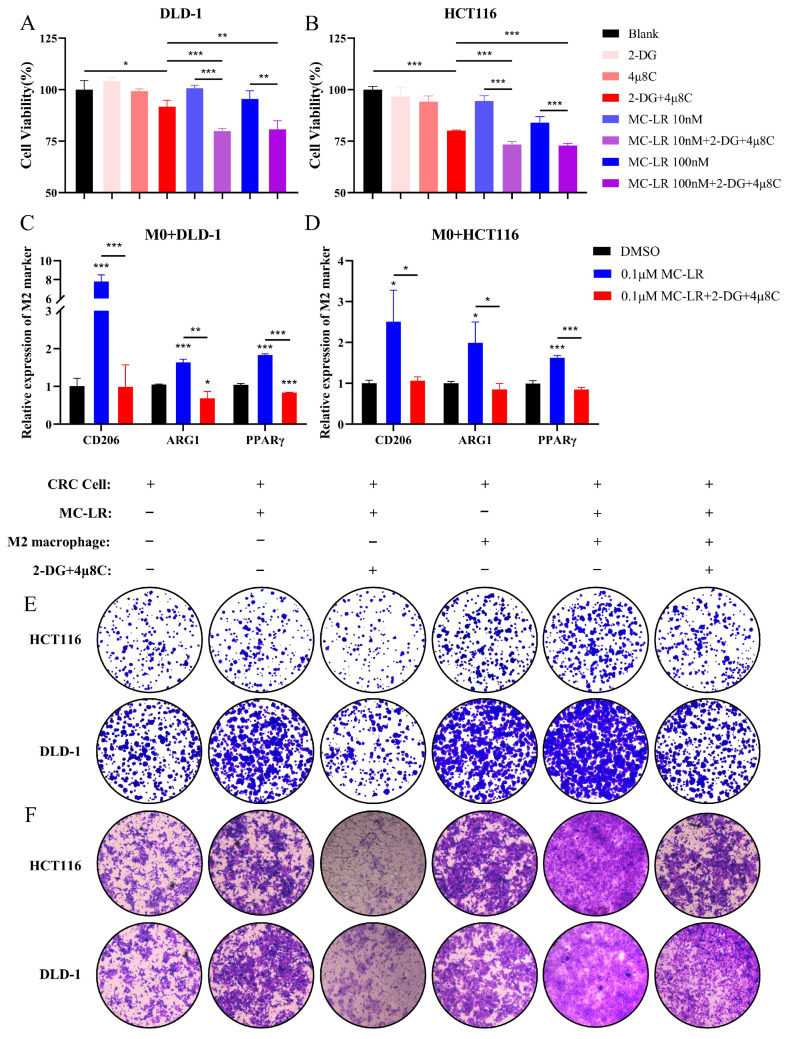
The combined treatment with 2-DG and 4μ8C suppressed the progression of CRC induced by MC-LR in both individual and co-culture culture conditions. Cell viability of (**A**) DLD-1 and (**B**) HCT116 cells was assessed under exposure to inhibitors (2-DG and 4μ8C), MC-LR (10 and 100 nM), and a combination of inhibitors (2-DG and 4μ8C) with MC-LR. 2-DG: 100μM; 4μ8C: 100nM. Relative expression levels of M2 markers (CD206, ARG1, PPARγ) in M0 macrophages co-cultured with (**C**) DLD-1 and (**D**) HCT116 cells, respectively, after 48 h exposure to MC-LR alone or in combination with inhibitors (2-DG and 4μ8C). (**E**) Colony formation and (**F**) Transwell migration of HCT116 and DLD-1 cells were evaluated after treatment with MC-LR alone or in combination with inhibitors (2-DG and 4μ8C) in co-culture and individual culture conditions. Original magnification, ×80. * *p* < 0.05, ** *p* < 0.01, *** *p* < 0.005.

Our results demonstrated that exposure of CRC cells to MC-LR promotes glycolysis via the IRE1α/XBP1/HK2 axis. We used 2-DG, a glycolysis inhibitor targeting hexokinase, and 4μ8C, an IRE1α/XBP1 pathway inhibitor, for further study. Initially, we assessed the EC50 values of 2-DG and 4μ8C on DLD-1 and HCT116 cells, individually (Figure S5). Subsequently, we co-treated CRC cells with 100 μM 2-DG and 100 nM 4μ8C, and observed a reduction in cell viability (Figure 7A,B). We further examined the impact of concurrent administration of 2-DG and 4μ8C on CRC cells following exposure to MC-LR (Figure 7A,B). The combined exposure increased the susceptibility of CRC cells, resulting in a notable reduction in cell viability compared to MC-LR exposure alone. Therefore, we used a lower concentration of MC-LR (0.1 μM) to examine the influence of this combined treatment on the M2 macrophage polarization within the co-culture environment of CRC cells and M0 macrophages following MC-LR exposure (Figure 7C,D). The findings indicated that co-administration of 2-DG and 4μ8C effectively suppressed M2 polarization of macrophages triggered by MC-LR exposure under co-culture settings involving M0 macrophages and CRC cells. Moreover, MC-LR exposure facilitated CRC cell migration and colony formation when M2 macrophages and CRC cells were co-cultured (Figure 7E,F). However, co-administration of 2-DG and 4μ8C suppressed this facilitation. The results indicate that the co-administration of 2-DG and 4μ8C inhibits MC-LR-induced communication between CRC cells and macrophages. This inhibition consequently impedes the progression of CRC, highlighting a potentially effective CRC target.

## 4. Discussion

MC-LR, as a potent carcinogen, presents substantial risks to human health. The extent to which it can facilitate the development of CRC via the regulation of the TME remains largely undisclosed. This study found that MC-LR had the ability to increase macrophage recruitment, inducing ERS in CRC cells to promote the M2 macrophage polarization. The crosstalk induced by MC-LR between macrophages and tumor cells facilitates the development of CRC.

In this study, CRC mice exposure to MC-LR significantly increased the number of infiltrating macrophages in tumor tissue, accompanied by adenoma-adenocarcinoma transformation, intestinal shortening, and increased angiogenesis. The macrophage infiltration may be linked to chemokines CXCL2, CXCL3, and CXCL5, released by CRC cells upon MC-LR exposure. In CRC, CXCL5 is predominantly released by cancerous epithelial cells, and its expression in CRC tissues is higher than in colon adenomas [54]. There exists a positive association between CXCL5 and macrophage infiltration within the TME [51]. Consistently, our findings demonstrated a direct relationship between CXCL5 and the macrophage presence in CRC. Moreover, there was a notable increase in the percentage of M2 macrophages. A recent study reported that ERS in tumor cells can be transferred to macrophages, with M1 macrophages exhibiting more cell death and M2 macrophages exhibiting less cell death in response to the same source of stress [55]. This difference may contribute to the increased proportion of M2 macrophages. Simultaneously, research has demonstrated that ERS is induced by MC-LR in liver cancer cells, as well as the NF-κB and TNFα signaling pathways, leading to the hepatotoxicity, inflammation, and carcinogenic effects [43]. Interestingly, our findings found that MC-LR triggered IRE1α/XBP1 pathway activation in CRC cells, both when cultured independently and when co-cultured with M0 macrophages. Furthermore, the IRE1α/XBP1 pathway activity within CRC cells can influence the extent of M2 macrophage polarization.

Chen et al. found that under hypoxic conditions, XBP1s regulate the HIF1a hypoxia response pathway to promote the development of triple-negative breast cancer, with HK2 being one of the regulated genes [56]. Similarly, silencing XBP1 reduces glioma cell viability by inhibiting HK2 expression, thereby regulating glycolysis [57]. This study predicted the binding site of XBP1s to the HK2 promoter and found that the IRE1α/XBP1 pathway activated by MC-LR promotes HK2 expression. HK2 is linked to the malignant progression and poor prognosis of CRC [58]. Therefore, we assessed lactate production and found that MC-LR exposure stimulates lactate via the IRE1α/XBP1/HK2 pathway, facilitating polarization of M2 macrophage. Earlier research has demonstrated that lactate-stimulated M2 macrophages enhance the angiogenesis, migration, and proliferation of breast cancer cells [59]. Our results demonstrate a significant promotion of CRC progression by M2 macrophages under MC-LR exposure. Moreover, the IRE1α/XBP1 pathway inhibitor 4μ8C and glycolysis inhibitor 2-DG significantly suppress MC-LR-induced polarization of M2 macrophage and the malignant progression of CRC cells. 2-DG is considered a promising anticancer agent, capable of enhancing the effects of various drugs [60,61]. 4μ8C can impede CRC cell proliferation at non-cytotoxic levels and augment the cytotoxic effect of 5-FU [62]. Therefore, combination therapy with both inhibitors may be a potential approach for treating CRC.

## 5. Conclusions

In summary, our results demonstrate that MC-LR enhances CRC progression through regulating the CRC cell–macrophage interaction. MC-LR activated the IRE1α/XBP1 signaling pathway in CRC cells, leading to increased expression of HK2, and promoted M2 polarization of macrophages via lactate as a mediator. The use of inhibitors 4μ8C and 2-DG effectively suppressed the migration and colony formation capabilities of CRC cells, and the M2 macrophage polarization in the co-culture environment under MC-LR exposure, providing potential target information for the treatment of CRC.

## Data Availability

Dataset available upon request from the authors.

## Supplementary Materials

The following supporting information can be downloaded at: https://www.mdpi.com/article/10.3390/cells13171439/s1, Original images can be found in Figure S1; Table S1: Primers for qRT-PCR.
