# Supplementary material for: Microcystin-LR Regulates Interaction between Tumor Cells and Macrophages via the IRE1α/XBP1 Signaling Pathway to Promote the Progression of Colorectal Cancer
Source: Cells. 2024 Aug 27;13(17):1439. doi: 10.3390/cells13171439 (PMC11394429; doi:10.3390/cells13171439)
Supplement: Supplementary file 1 [file cells-13-01439-s001.zip › Table S1.pdf]

Supplementary Table 1. Primers for qRT-PCR

Gene	Seqences
human GRP78	F: CATCACGCCGTCCTATGTCG
	R: CGTCAAAGACCGTGTTCTCG
human IRE1α	F: CCTGCAGGACTGGATCTTCT
	R: CGGCCTCGGGATTTTTGGAA
human XBP1s	F: CCTGGTTGCTGAAGAGGAGG
	R: CCATGGGGAGATGTTCTGGAG
human HK2	F: AAGGCTTCAAGGCATCTG
	R: CCACAGGTCATCATAGTTCC
human CD206	F: CTACTGAACCCCCACAAC
	R: AAACCAGAGAGGAACCCA
human Arg1	F: CTGTGGGAAAAGCAAGCGAG
	R: CATGGCCAGAGATGCTTCCA
human PPARγ	F: ACCAAAGTGCAATCAAAGTGGA
	R: ATGAGGGAGTTGGAAGGCTCT
human INOS	F: AGGGACAAGCCTACCCCTC
	R: CTCATCTCCCGTCAGTTGGT
human CD80	F: AAACTCGCATCTACTGGCAAA
	R: GGTTCTTGTACTCGGGCCATA
human β-actin	F: GCGAGAAGATGACCCAGATC
	R: CCAGTGGTACGGCCAGAGG
mouse CXCL2	F: CACCAACCACCAGGCTAC
	R: TCAGGGTCAAGGCAAACT
mouse CXCL3	F: TCTCACCACAGCCCTTCGCA
	R: AAAAACAAGCAGGTAAAGACACATC
mouse CXCL5	F: GTTCCATCTCGCCATTCATGC
	R: GCGGCTATGACTGAGGAAGG
mouse β-actin	F: TCAAGATCATTGCTCCTCCTGAG

R: ACATCTGCTGGAAGGTGGACA
