# Supplementary material for: Microcystin-LR Regulates Interaction between Tumor Cells and Macrophages via the IRE1α/XBP1 Signaling Pathway to Promote the Progression of Colorectal Cancer
Source: Cells. 2024 Aug 27;13(17):1439. doi: 10.3390/cells13171439 (PMC11394429; doi:10.3390/cells13171439)
Supplement: Supplementary file 1 [file cells-13-01439-s001.zip › Weatern blot .pptx]

## Slide 1
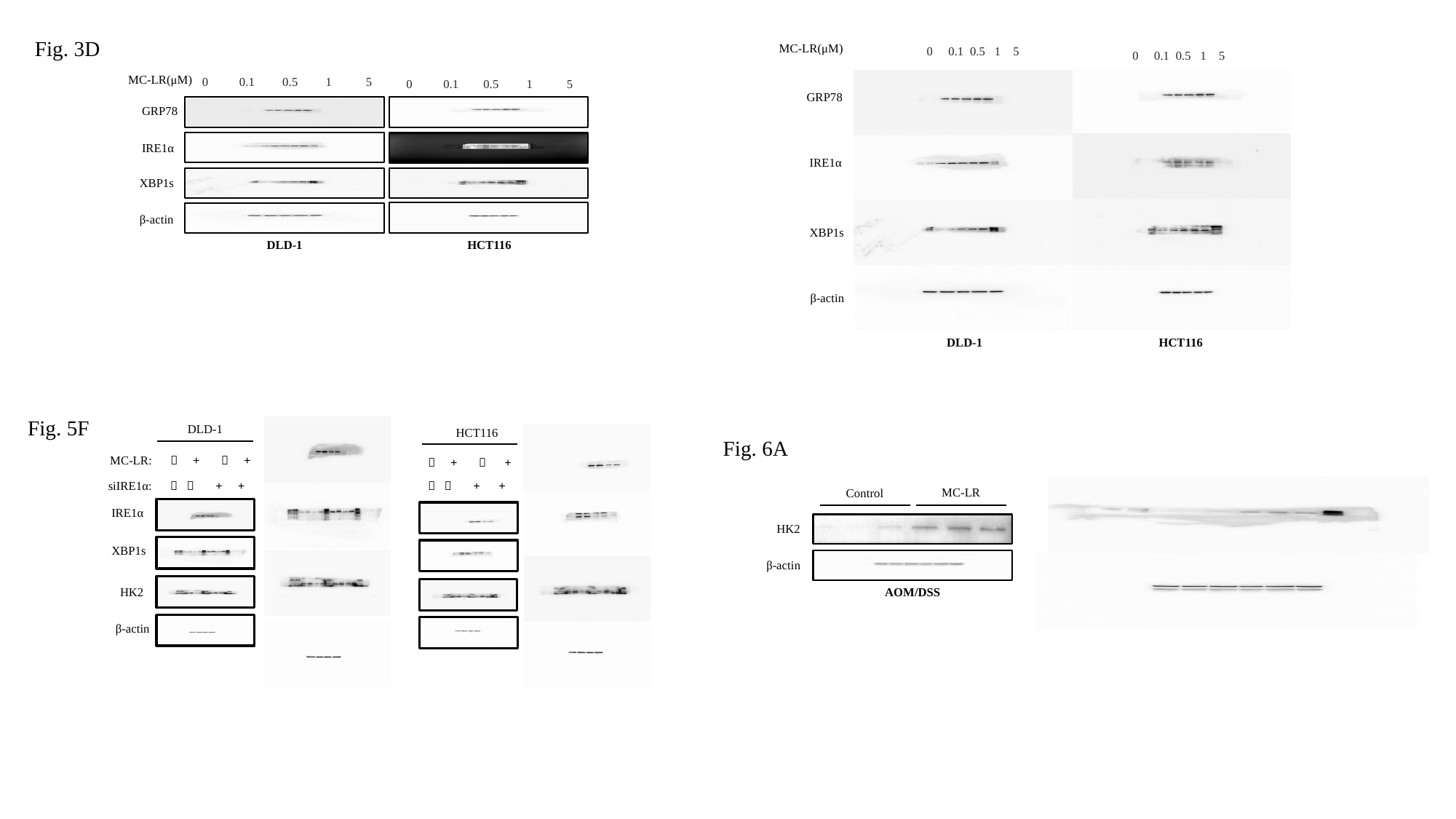

Fig. 3D
 0 0.1 0.5 1 5
MC-LR(μM)
 0 0.1 0.5 1 5
 0 0.1 0.5 1 5
MC-LR(μM)
 0 0.1 0.5 1 5
GRP78
GRP78
IRE1α
IRE1α
XBP1s
β-actin
XBP1s
HCT116
DLD-1
β-actin
DLD-1
HCT116
Fig. 5F
DLD-1
HCT116
Fig. 6A
MC-LR: － + － +
－ + － +
siIRE1α: － － + +
－ － + +
MC-LR
Control
IRE1α
HK2
XBP1s
β-actin
HK2
AOM/DSS
β-actin
